# The novel transcriptional factor HP1BP3 negatively regulates *Hsp70* transcription in *Crassostrea hongkongensis*

**DOI:** 10.1038/s41598-017-01573-y

**Published:** 2017-05-03

**Authors:** Delin Xu, Qin Yang, Miao Cui, Qizhong Zhang

**Affiliations:** Department of Ecology, Institute of Hydrobiology, School of Life Science and Technology, Key Laboratory of Eutrophication and Red Tide Prevention of Guangdong Higher Education Institutes, Engineering Research Center of Tropical and Subtropical Aquatic Ecological Engineering, Ministry of Education, Jinan University, Guangzhou, 510632 P.R. China

## Abstract

*Ch*HP1BP3, a chromatin complex-related protein known with dynamic features, was identified as a *ChHsp70* promoter-associated factor in *Crassostrea hongkongensis* by DNA-affinity purification and mass spectrometry analysis. Direct interaction between purified *Ch*HP1BP3 and the *ChHsp70* promoter region was demonstrated using EMSA. *ChHp1bp3* depletion led to clear enhancements in *ChHsp70* mRNA expression in *C*. *hongkongensis* hemocytes. However, *ChHp1bp3* overexpression in heterologous HEK293T cells correlated with fluctuations in *ChHsp70* transcription. Quantitative RT-PCR analysis showed that both *ChHsp70* and *ChHp1bp3* transcription were responsive to external physical/chemical stresses by heat, CdCl_2_ and NP. This indicated a plausible correlation between *ChHsp70* and *ChHp1bp3* in the stress-induced genetic regulatory pathway. While, the distinctive *ChHp1bp3* expression patterns upon physical and chemical stresses suggest that the mechanisms that mediate *ChHp1bp3* induction might be stress-specific. This study discovered a novel role for HP1BP3 as a negative regulator in controlling *Hsp70* transcription in *C*. *hongkongensis*, and contributed to better understanding the complex regulatory mechanisms governing *Hsp70* transcription.

## Introduction

Hsp70s are a group of stress-inducible heat shock proteins that are approximately 70 kDa^1^. They are highly conserved molecular chaperones that are universally present in all cellular species and play critical roles in assisting the three-dimensional folding of newly synthesized proteins and restoring disordered proteins caused by a variety of intense stresses, including heat shock, heavy metals, and toxic chemicals^2, 3^. Hsp70 is the largest and currently the most extensively studied stress protein family. Owing to its chaperone activity as well as its importance for allowing cells to cope with stress insults, Hsp70 has been used for many applications in various disciplines, including protecting against and therapeutic treatments for cardiovascular diseases, cancer prognosis, aiding in transmembrane protein transport, inhibiting apoptosis, and biomonitoring^4–6^. To further explore potential applications of Hsp70s, a comprehensive understanding of the mechanisms governing their response to various stresses is demanded.

*Hsp70* activiation has been primarily investigated at the transcriptional level. Numerous studies have shown that *Hsp70* transcription undergoes integrated regulation from both general and gene-specific regulators, including GAGA factor, TBP (TATA-binding protein), STAT (signal transducer and activator of transcription), and HSF-1 (heat shock factor 1)^7–9^. Heat-shock activation of *Hsp70* is also believed to be regulated by promoter-proximal pausing, a rate-limiting step prior to productive transcription^10, 11^. The stall is regarded as a regulatory checkpoint; the transition of promoter-proximal Pol II into productive elongation is triggered by HSF binding signals to allow rapid and synchronous activation of *Hsp70* ^12^. The overall dynamic architecture of chromatin is critical in regulating gene expression levels, since increased chromatin dynamics facilitate factor recruitment to DNA, thus promoting activation^13–15^. Multiple interdependent regulators such as the TFIID and GAGA factor have been shown to manipulate nucleosome pattern and subsequently affect *Hsp70* promoter architecture, most likely through associations with NURF (nucleosome remodeling factor) and dFACT (facilitates chromatin transcription)^10, 16, 17^. Although heat shock response is regarded as the gold standard for investigating gene regulation, the full details of its induction pathway remain incomplete.

In aquatic animals, the evaluation of stress protein expression levels has been advocated as a distinctive environmental monitoring tool^18^. *Hsp70* is thought to be favorable for biomonitoring owing to the extreme sensitivity of the *Hsp70* gene *per se* to various aquatic pollutants^19^. Oysters are considered as ideal monitoring hosts due to numerous advantageous attributes of the animals, such as sessile living, tolerance to various pressures, abundance, long lifespan, and sufficient tissue mass for analysis. *Hsp70* expression in oysters has been reported to be responsive to a variety of environmental stimuli, including heat shock and various inorganic (heavy metals, salinity etc.) and organic (antifouling biocides) chemicals^20–24^. Despite its environmental and biological importance, the mechanisms controlling *Hsp70* transcription induction in oysters remain largely unknown, although eight HSF-1 isoforms have been identified to be inducible by hypoxia in Pacific oysters^25^. In previous studies, we have isolated *ChHsp70* promoter-bound proteins from nuclear extracts prepared from *Crassostrea hongkongensis* either with or without stress treatment^26^. However since different protein patterns were shown in DNA-affinity purification upon various stressed conditions, it was proposed that the differences are caused either by the distinct regulatory proteins showing affinity to the promoter or by dynamic interactions of specific factors from the same batch of regulators. In this study, heterochromatin protein 1 binding protein 3 (HP1BP3) was isolated after a prolonged incubation by DNA-affinity purification. A specific interaction between *Ch*HP1BP3 and the *ChHsp70* promoter region was discovered *in vitro* and a negative regulatory function of *Ch*HP1BP3 on *ChHsp70* transcription was demonstrated in its native host. The different *ChHp1bp3* induction patterns shown by RT-PCR indicated that different mechanisms govern *ChHP1BP3* induction upon physical and chemical stresses.

## Results

### Identification of proteins showing dynamic affinity to the *ChHsp70* promoter

Our previous results of DNA-affinity purification demonstrated distinctive patterns for regulators showing affinity to the *ChHsp70* promoter region under stressed or non-stressed conditions. To discover the possible involvement of putative regulators bearing dynamic binding activities, DNA affinity purification was performed with either 15 min or 30 min protein-DNA incubation times. The results are shown in Supplementary Fig. 1. When comparing the banding patterns of the two groups, most of the proteins were consistently isolated in either the control or stressed sample lanes. However, there were some protein bands that specifically appeared in the 30 min incubation stressed lanes, including one that was noticeably visible right above the 45 kDa marker. The nature of the latter band was revealed by mass spectrometry as HP1BP3 (Supplementary Fig. 2). The time- and stress- dependent affinity of HP1BP3 to the *ChHsp70* promoter was of interest and was thus selected for further study.

### Characterization of the *ChHp1bp3* cDNA

**Figure 1:**
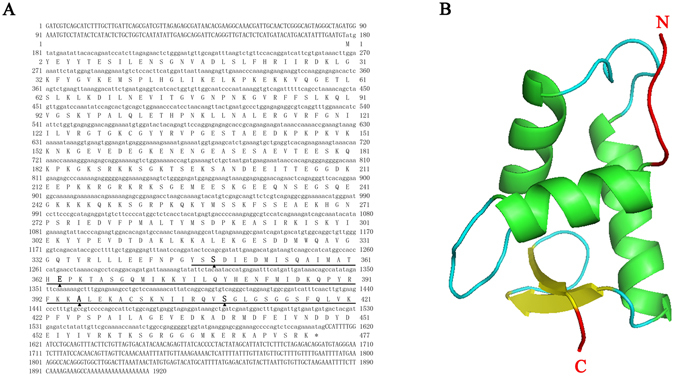
(**A**) The cDNA sequence and deduced amino acid sequence of *Ch*HP1BP3 in *C*. *hongkongensis*. The nucleotides and amino acids are numbered along both margins. The region of globular domain is underlined. The putative DNA binding sites are marked by triangles. The translation stop codon is indicated with an asterisk (*). (**B**) Three dimensional structure of the globular domain of *Ch*HP1BP3.

The *ChHp1bp3* cDNA is 1920 bp in length and contains a 1434 bp coding region as well as 177 bp and 309 bp 5′ and 3′ untranslated regions, respectively (Fig. 1A). The peptide sequence is composed of 477 amino acids, with a molecular weight of 53.7 kDa and an isoelectronic point of 9.19. A globular domain, which has been previously reported to be structurally related to that of histone H1 and interact with nucleosomal DNA^27^, was discovered at the C-terminal region of the *Ch*HP1BP3 peptide sequence (Fig. 1A). The amino acid sequence of the globular domain was then subjected to three dimensional structure prediction using the online SWISS-MODEL software (http://swissmodel.expasy.org/), and the deduced protein model revealed the presence of three α–helices and two β–sheets (Fig. 1B).

### *Ch*HP1BP3 directly interacts with the *ChHsp70* promoter region *in vitro*

**Figure 2:**
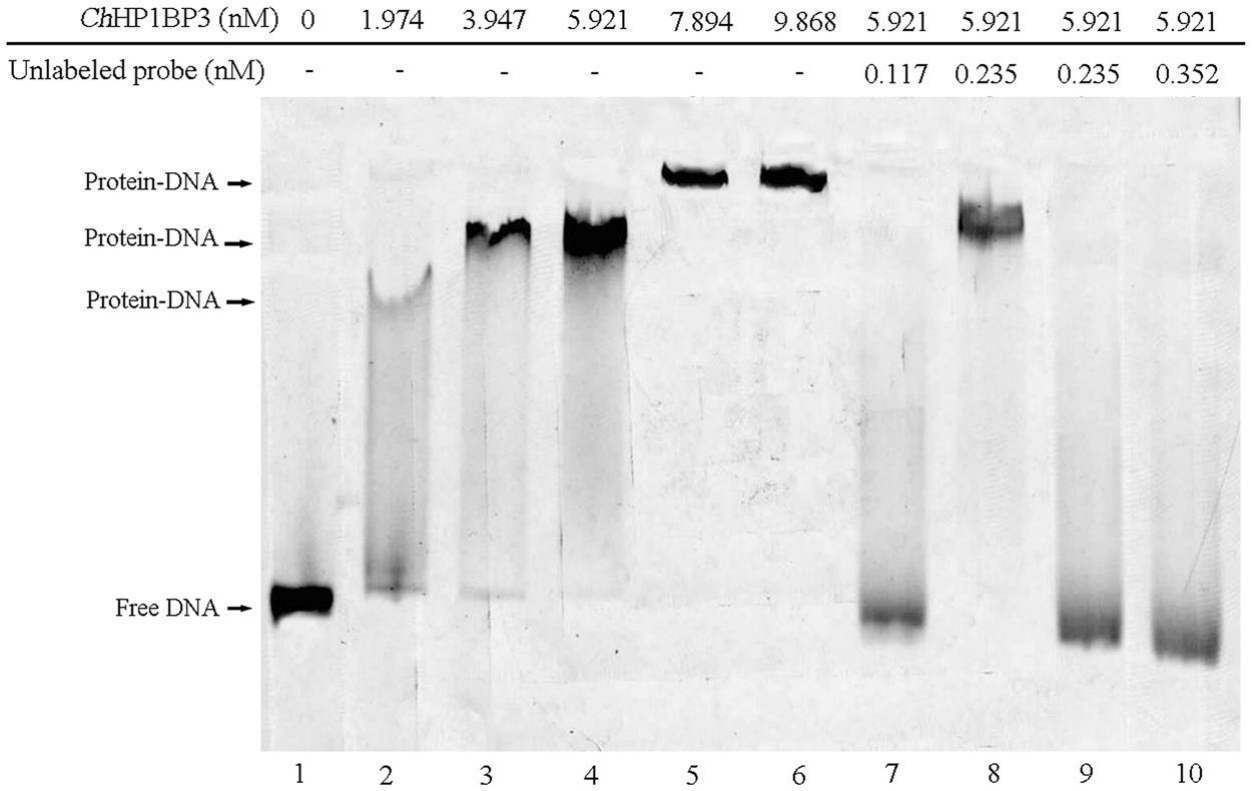
Electrophoretic mobility shift assay of the *ChHsp70* promoter with purified His_6_-*Ch*HP1BP3. Fifty picomoles of FITC-labeled *ChHsp70* promoter was added to all the lanes. The band positions of the shifted DNA-protein complexes and free DNA are indicated by arrows.

The ability of *Ch*HP1BP3 to directly interact with the *ChHsp70* promoter region was tested via EMSA using a FITC-labeled *ChHsp70* promoter region and His_6_-*Ch*HP1BP3 purified from *Escherichia coli*. The results in Fig. 2 clearly demonstrated that the addition of His_6_-*Ch*HP1BP3 delayed the electrophoretic mobility of the *ChHsp70* promoter region in a concentration dependent manner (Fig. 2, lanes 2 to 6). A competition assay was performed by introducing in excess amounts of unlabeled *ChHsp70* promoter to the reaction mixture, which successfully out-competed the specific interactions, thus eliminating the presence of the shifted bands (Fig. 2, lanes 7, 9 and 10). However, when an irrelevant DNA fragment (+370 to +763 relative to the translational start on the *ChHp1bp3* coding sequence) was introduced, the competition was largely compromised (Fig. 2, lane 8). These results indicated a specific interaction between His_6_-*Ch*HP1BP3 and the *ChHsp70* promoter region.

### *ChHp1bp3* knockdown enhanced *ChHsp70* transcription in *C. hongkongensis* hemocytes

**Figure 3:**
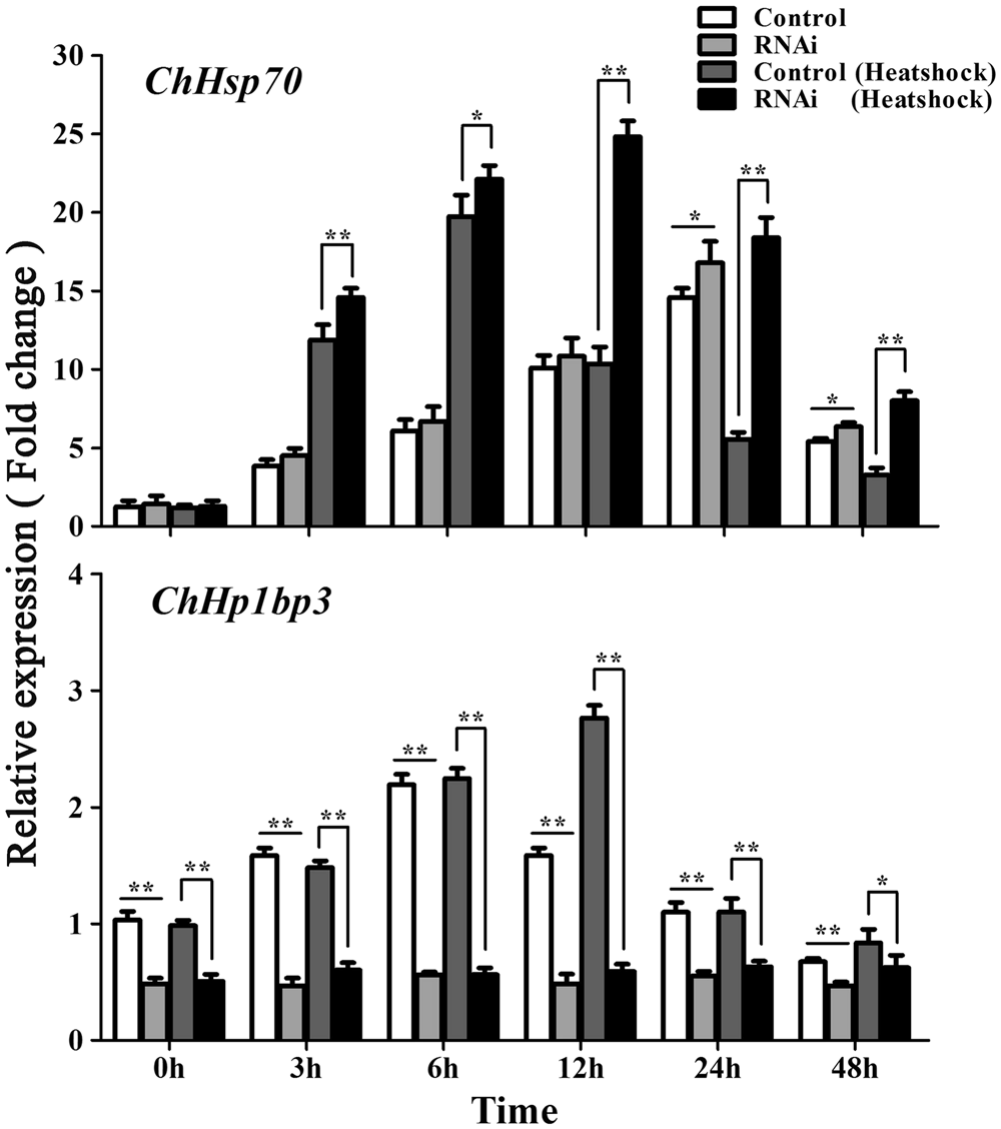
*ChHsp70* and *ChHp1bp3* expression levels by RT-PCR after the addition of either control RNA (Control) or siRNA (RNAi). All values are expressed as the means ± SD (n = 3). Asterisks above the bars indicate that values are significantly different from the individual controls (*P < 0.05 and **P < 0.01).

To discover the regulatory functions of *Ch*HP1BP3 on *ChHsp70* transcription in either basal or heat shocked condition, *ChHsp70* mRNA expression was measured by RT-RCR in both wild-type and siRNA-treated hemocytes of *C*. *hongkongensis* as described in the Materials and methods. The results are shown in Fig. 3. *ChHp1Bp3* mRNA expression was significantly depleted at all the tested time points either with or without heat shock treatment when the cells were treated with siRNA, validating the successful knockdown of *ChHp1bp3* expression. *ChHsp70* basal transcription was clearly enhanced particularly at 24 and 48 h upon *ChHp1bp3* depletion. Heat induced *ChHsp70* expression was shown to be more sensitive to *ChHp1bp3* levels since more significant enhancement was observed to the already induced *ChHsp70* transcription when *ChHp1bp3* was depleted, and the effective impact was visible nearly at all the tested time points (3 h, 6 h, 12 h, 24 h and 48 h). These results demonstrate a negative role for *Ch*HP1BP3 in regulating *ChHsp70* transcription under either basal or heat-stressed conditions in native *C*. *hongkongensis* hosts. Noticeably, an increasing expression profile was presented by the basal *ChHsp70* transcription until 24 h. This induction is likely due to the experienced stress caused by altered biological context during the *in vitro* culture of the hemocytes.

### *Ch*HP1BP3 overexpression manipulates *ChHsp70* expression in heterologous host HEK293T cells

**Figure 4:**
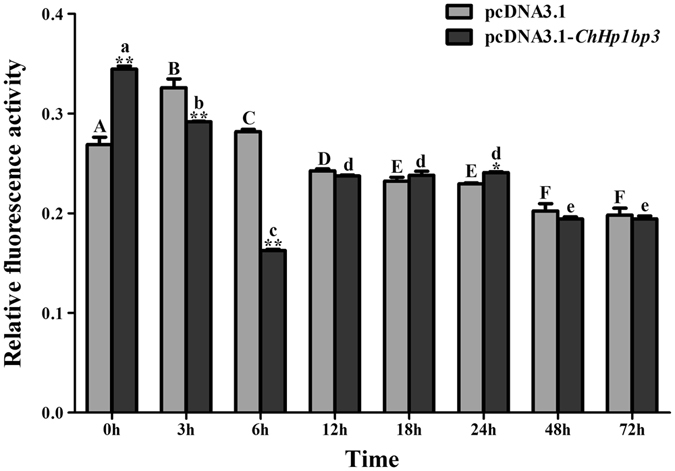
Luciferase activity driven by the *ChHsp70* promoter in HEK293T cells following the addition of pcDNA3.1-*ChHp1bp3* and pcDNA3.1. All values are presented as the means ± SD (n = 3). Asterisks above the bars indicate the values are significantly different from the individual controls. (*P < 0.05 and **P < 0.01). Bars on the same group with different letters are statistically significant from one another.

A luciferase reporter assay was conducted in HEK293 cells to discover the regulatory effects of overexpressed *Ch*HP1BP3 on *ChHsp70* expression. The results are shown in Fig. 4. *ChHsp70* promoter activity was notably enhanced by overexpressed *Ch*HP1BP3 at the first sampling point (24 h post transfection), indicating an initial positive regulatory role for *Ch*HP1BP3 on *ChHsp70* transcription. However, this activated state was soon overturned since a clear decrease in *ChHsp70* promoter activity was observed at 3 h and 6 h. *ChHsp70* transcription recovered from 12 h until it reached a level comparable to the control at 18 h. Interestingly, promoter activation was once again shown at 24 h, whereas the induced state was totally compromised from 48 h onwards. These results demonstrated that constitutively expressed *Ch*HP1BP3 caused *ChHsp70* expression to fluctuate, meaning that *Ch*HP1BP3 regulated *ChHsp70* expression in an oscillating manner. This suggests that a complex mechanism underlies *Ch*HP1BP3 controlled *ChHsp70* transcription in the heterologous HEK293T host.

### *ChHsp70* and *ChHp1bp3* transcription profiles upon various stresses

**Figure 5:**
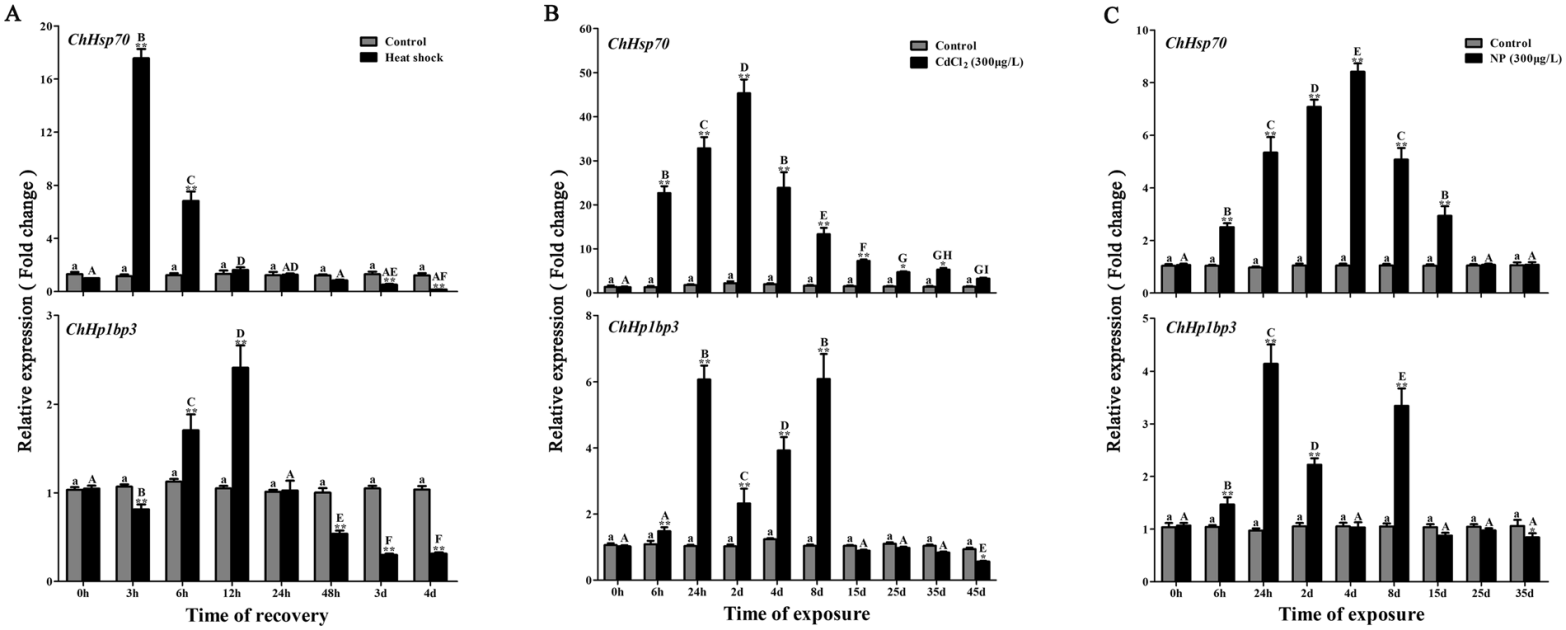
*ChHsp70* and *ChHp1bp3* expression levels by RT-PCR after (**A**) heat, (**B**) CdCl_2_ or (**C**) NP stress. All values are expressed as the means ± SD (n = 3). Asterisks above the bars indicate the values are significantly different from the individual controls. (*P < 0.05 and **P < 0.01). Bars on the same group with different letters are statistically significant from one another.

In an attempt to reveal correlations between *ChHsp70 and ChHp1bp3* transcription in response to external stresses, quantitative real-time PCR was employed to determine *ChHsp70 and ChHp1bp3* mRNA expression in oysters gills treated with heat, CdCl_2_ or 300 μg/L NP, which has been previously determined as a suitable concentration for induction^26^. The results are shown in Fig. 5. *ChHsp70* transcription was notably induced upon stresses, with the highest peaks induced by heat, Cd and NP at 3 h, 2 d, and 4 d, respectively. After that, *ChHsp70* expression declined gradually for all three treatments until it reached a level comparable to the controls. *ChHp1bp3* transcription was induced by all the tested stresses. However, a different induction pattern was observed for CdCl_2_ and NP treatment since two different induction peaks were shown. The two peaks were accumulated at the time points of 24 h and 8 d, which either preceded or followed peak induced *ChHsp70* transcription, respectively. Upon heat stress, a single *ChHp1bp3* induction peak was shown at 12 h; this peak was postponed for 9 h compared to the *ChHsp70* peak. These results demonstrated that both *ChHsp70* and *ChHp1bp3* were transcriptionally responsive to external stresses, though in distinctive patterns. This indicated a plausible correlation between the two genes in a genetic regulatory pathway induced by stresses.

## Discussion

Heterochromatin, a highly compacted structure of higher-order chromatin, is formed dynamically and alternately during interphase, during which most chromatin-dependent biological events occur. The non-histone proteins that temporally interact with chromatin during interphase are considered important for elucidating a variety of host cell functions^28–30^. HP1BP3 has been revealed to be a chromatin complex associated protein with strikingly dynamic features during the distinct stages of interphase, demonstrating that it plays critical and diverse roles in various cellular processes, including heterochromatin organization, nucleosome assembly, cell proliferation regulation, cellular response to hypoxia, and DNA-templated transcription regulation^31–33^. In the present paper, we reported the characterization of an HP1BP3 homologue *Ch*HP1BP3 in *C*. *hongkongensis*. It directly interacts with the *ChHsp70* promoter *in vitro*, and its depletion was shown to activate *ChHsp70* transcription in the native host, and its overexpression caused *ChHsp70* transcription to fluctuate in the heterologous host HEK293T.

Analysis of the *Ch*HP1BP3 amino acid sequence revealed the presence of a globular “Histone H-1 like” domain (Fig. 1A), which is present in other HP1BP3 homologues^27^. This domain was reported to confer DNA binding characteristics^34^. Indeed, our EMSA results demonstrated a clear mobility shift by the *ChHsp70* promoter upon the addition of *Ch*HP1BP3. However, the shifted bands migrated more slowly when His_6_-*Ch*HP1BP3 was introduced at higher concentrations (Fig. 2). This result raised the possibility of the presence of multiple *Ch*HP1BP3 binding sites in the *ChHsp70* promoter region or the formation of His_6_-*Ch*HP1BP3 multimers that interact with a core binding site.

RNAi analysis demonstrated that *ChHsp70* mRNA expressions was clearly induced by *Ch*HP1BP3 depletion under either basal or heat-stressed conditions compared to the control (Fig. 3). Taken together with the above EMSA data, these results suggest that *Ch*HP1BP3 negatively regulates *ChHsp70* transcription via direct interaction with its promoter region. HP1BP3 has been previously characterized as a histone H1 related protein that associates with chromatin by binding to nucleosome surfaces and interacting with nucleosomal DNA^27^. These proteins are considered highly important for transcription regulation since they promote chromatin high-order structure, which has been widely shown to restrict the transcriptional machinery access to the template DNA, thus fundamentally determining host cell transcriptional activities. Indeed, some chromatin structure maintaining proteins have been shown to act as transcriptional regulators to control the expression of specific genes or gene clusters. For example, Butchko *et al*. reported the global regulatory role of LaeA on the expression of multiple secondary metabolite gene clusters by altering heterochromatin structure in cooperation with the velvet complex in fungi^35^. To confirm whether *Ch*HP1BP3 affects *C*. *hongkongensis* chromatin structure, chromatin was extracted from both control and *ChHp1bp3-*depleted oyster hemocytes and subjected to partial MNase digestion using the methods described in the Supplementary data. The digestion patterns of the chromatin extracted from the hemocytes 24 h post *ChHp1bp3* knockdown are shown as a representative in Supplementary Fig. 3. Compared to the control cells, the *ChHp1bp3*-depleted chromatin samples were always more susceptible to MNase digestion in either non-stressed or heat-stressed conditions. This suggests that *Ch*HP1BP3 may indeed be required for the formation of higher-order chromatin structure. Thus, *Ch*HP1BP3 depletion is expected to unpack heterochromatin and disrupt its structure, leading to the regulation of numerous DNA-dependent events including transcription. Therefore, the discovered negative regulatory role of *Ch*HP1BP3 on *ChHsp70* expression might also be a result of heterochromatin structure alterations. Taken together with the above EMSA data, it is reasonable to propose that the observed negative regulation on *ChHsp70* transcription might be due to a coordinated contribution from *Ch*HP1BP3 activities on both pathway-specific regulation and global chromatin structure change. The regulatory roles of HP1BP3 on the expression of various molecular chaperones have been previously reported in 293T by iTRAQ analysis. Though inconsistently, Hsp70 was found to be positively regulated^31^. This discrepancy suggests that HP1BP3 may interact in a host-dependent manner for controlling *Hsp70* expression, which plausibly involves distinct regulatory mechanisms. Considering that several general (GAGA factor, TBP, etc.) and gene-specific (HSF-1, GAF, etc.) regulators have been previously discovered to control *Hsp70* transcription either cooperatively or competitively, determining plausible partner regulators that interplay with *Ch*HP1BP3 on the *ChHsp70* promoter region is highly relevant to better understand mechanisms that involve *Ch*HP1BP3 and govern *ChHsp70* transcription.

Since *Ch*HP1BP3 depletion had a positive effect on *ChHsp70* expression, it was thought that conversely, *Ch*HP1BP3 overexpression might correlate with a repressive phenotype. However, while clear repressions were indeed shown at various sampling points upon *Ch*HP1BP3 overexpression by the luciferase reporter assay, an overall fluctuating regulatory effect was demonstrated in the heterologous HEK293T cells. It has been previously noted that the association of HP1BP3 with the chromatin complex is strikingly dynamic^31^. This typical feature allowed us to hypothesize that the unusual fluctuations might be due to intermittent regulation by the overexpressed *Ch*HP1BP3 that dynamically interacted with the *ChHsp70* promoter. Fortunately, this notion was supported by our DNA affinity purification result, which demonstrated a time-dependent affinity between *Ch*HP1BP3 and the *ChHsp70* promoter (Supplementary Fig. 1). The reason for the strong induction of *ChHsp70* expression at the first time point was unknown, but considering that the expression of hundreds of genes could be affected by HP1BP3 depletion, it is reasonable to expect an initial emergence of any activator induced by overexpressed *Ch*HP1BP3 in the heterologous host cells.

*Hsp70* has been widely acknowledged as a valid bioindicator to signify environmental stresses since its expression is extremely sensitive to various stimulants^19^. In our previous studies, the expression of *ChHsp70* of *C*. *hongkongensis* has been demonstrated to be significantly induced by both heat and Cd stress^26^. Consistent with these data, similar induction profiles demonstrating induction accumulation, peaking, a gradual decline and recovery were shown by RT-PCR analysis upon the two stresses as well as by NP treatment. NP is a notorious endocrine disruptor that is frequently found but not readily biodegradable in aquatic environments, meaning that it poses continuous hazards to aquatic animals and human health^36^. *ChHp1bp3* transcription was also induced by all three stresses, though different induction patterns were observed. The second accumulated *ChHp1bp3* expression peaks induced by Cd and NP together with the single heat-induced peak appeared at points that were slightly delayed when compared with the corresponding *ChHsp70* peaks. Considering the previously discovered negative regulatory function, these late accumulated *ChHp1bp3* peaks might be very likely to contribute to the recovery process for promoting *ChHsp70* expression back to its original level. An additional earlier *ChHp1bp3* induction peak was present upon either Cd or NP stress compared with the *ChHsp70* peak. It is currently unknown whether this early peak correlates directly with *ChHsp70* induction based on our current data. However, its absence upon the heat stressed condition indicates that different mechanisms govern *ChHp1bp3* induction following physical and chemical stress.

In summary, we demonstrated a direct interaction between *Ch*HP1BP3 and the *ChHsp70* promoter region, and a clear negative regulatory role for *Ch*HP1BP3 on *ChHsp70* transcription in *C*. *hongkongensis*. The differences in the *ChHp1bp3* induction patterns due to various external stresses as well as the discrepancies in the *Ch*HP1BP3 regulatory effects on *ChHsp70* transcription in a heterologous HEK293T host cells suggest that HP1BP3 mediated regulation is rather complex and likely involves the cooperation of additional factors. This study provided novel insights into HP1BP3 mediated transcriptional regulation and presents the first evidence of a specific HP1BP3 regulatory target gene.

## Materials and Methods

### Oyster collection, maintenance and treatment

Oysters (*C*. *hongkongensis*) were collected from a farm located in the Yangxi suburb, in Guangdong, China. All of the sampled oysters were one-year old and had a mean shell length of 9 ± 1 cm. The oysters were transferred to Jinan University within 3 h and allowed to acclimate for 2 weeks at 24 ± 1 °C in aerated artificial seawater with 16~18‰ salinity. The oysters were fed algal slurry once a day under a natural photoperiod. The seawater was renewed every other day. After the preliminary acclimation, the oysters were randomly picked and evenly transferred to 25 L plastic tanks filled with artificial sea water supplemented with either CdCl_2_ or nonylphenol (NP) at 300 µg/L; the tanks without any supplements were used as the control. The seawater was renewed every other day with the initial CdCl_2_ or NP concentrations. For thermal stress, the acclimated oysters were transferred to a 37 °C water bath and maintained for 1 h, followed by recovery under the original conditions for 3 h.

### DNA affinity purification

DNA affinity purification was performed as previously described^26^ but with slight modifications. Briefly, biotin-labeled *ChHSP70* promoter was amplified from *C*. *hongkongensis* genomic DNA using a primer pair of P70-promoter up and P70-promoter down; the PCR product was subsequently immobilized to streptavidin-coated magnetic beads (Cat. No. 88816; Thermo) in DNA-binding buffer (10 mM Tris-HCl, 1 mM EDTA, and 2 M NaCl, pH 7.5) in accordance with the manufacturer’s instructions. After washing, the DNA coated beads were resuspended in protein-binding buffer (20 mM Tris-HCl, 4.5 mM EDTA, 60 mM NaCl, 10 mM HEPES, 5 mM CaCl_2_, 50 mM KCl, 9% sucrose (w/v), and 12% glycerol, pH 7.5). Then nuclear protein extracts that were previously prepared from gills of the thermal/Cd stressed and the control oysters^26^ were individually introduced, and the reaction mixture was incubated at room temperature for either 15 min or 30 min. A second washing step was performed using protein binding buffer, and then the proteins showing affinity to the *ChHsp70* promoter were eluted with elution buffer (25 mM Tris-HCl and 200 mM NaCl, pH 7.5). The eluted proteins were then analyzed by SDS-PAGE with Coomassie Brilliant Blue staining. The individual bands of interest were identified by mass spectrometry as described in the supplementary materials.

### Full-length *ChHp1bp3* cDNA sequencing

Total RNA was extracted from oysters gills using the Trizol reagent (Cat. No. 15596-026; Invitrogen) following the manufacturer’s instructions. The integrity and concentration/purity of the isolated RNAs were subsequently examined by agarose gel electrophoresis and spectrometry analysis, respectively. First strand cDNA was synthesized from two micrograms of total RNA at 55 °C for 30 min with 200 U M-MLV Reverse Transcriptase (Promega) using oligo (dT) primer. The resulting cDNA pool was then applied as template to amplify the *ChHp1bp3* coding sequence with the HP1BP3-up and HP1BP3-down PCR primers (Table 1). The amplified products were cloned into the pMD18-T (TaKaRa) vector, and the inserted *ChHp1bp3* coding sequence was revealed by nucleotide sequencing (Liuhe BGI).

**Table 1:** Synthetic oligonucleotides used in this study.

Primer	Sequence 5′ → 3′	Position(s)	Purpose
Oligo(dT)18	TTTTTTTTTTTTTTTTT		Reverse transcription
P70-promoter up	TTCCATGCACTCTAGCTAAG	−304–285	Amplification of *ChHsp70* promoter fragment for DNA-affinity purification and EMSA
P70-promoter down	GAATCGTAGTCTAGTTGACTC	+40–+60	
HP1BP3-up	ACATATTTGAATGTATGTATGAAT	−14–+10	Amplification of *ChHp1bp3* coding sequence
HP1BP3-down	ACTGTTGTGGATAAAGATTCCCT	+1528–+1550	
UP	CGGCAGTGGTATCAACGCAGAGTAC		Universal primer used with 3GSP2 and 5GSP2
UP(dG)	CGGCAGTGGTATCAACGCAGAGTAC(G)10		5′ RACE PCR
5GSP1	TATTTCTTCATCATTAGCA	+594–+612	
5GSP2	TTCCCATGTTTTTCCGCCTCTGAC	+789–+812	
UP (dT)	CGGCAGTGGTATCAACGCAGAGTAC(T)18		3′ RACE PCR
3GSP1	TACATAGAAAAGTATTAC	+898–+915	
3GSP2	TCAGATGACATGTGGCAGGCTGTT	+967–+990	
HP1BP3-F-NdeI	GGGAATTCCATATGTATGAATAT	+1–+12	Amplification of *ChHp1bp3* for protein expression
HP1BP3-R-XhoI	CCGCTCGAGTTTTCTGGAGAC	+1420–+1431	
HP1BP3-E-KpnI	ATAAAAGGTACCATGTATGAATATTACACAGAATCCATC	+1–+27	Amplification of *ChHp1bp3* for overexpression
HP1BP3-E-XhoI	ATAAAACTCGAGCTATTTTCTGGAGACTGGGGC	+1414–+1434	
Actin-Up	CTGTGCTACGTTGCCCTGGACTT	+701–+723	RT-PCR
Actin-Down	TGGGCACCTGAATCGCTCGTT	+829–+849	
RT-HP1BP3-Up	AAACAGCCTCAGGACAGATG	+1094–+1113	RT-PCR
RT-HP1BP3-Down	CAAGTTGAAATGATCCGCCAC	+1238–+1258	
RT-HSP70-Up	GCCAAACTACATCAGAACGGGTC	−1860–1838	RT-PCR
RT-HSP70-Down	TCCATCTCCTCTACAGTCGGTCC	−2461–2439	

Two micrograms of total RNA was used for reverse transcription with 2 pmol of UP (dT) and 200 U M-MLV Reverse Transcriptase; the amplified products were subsequently purified using a QIA quick column (Qiagen). A homopolymeric C tail was added to the 3′-end of the cDNA using terminal deoxynucleotidyl transferase (TaKaRa) according to the manufacturer’s instructions. Then, the sample was purified again by QIA quick column (Qiagen). The resultant poly (dC)-tailed cDNA was then applied as a template for the PCR reaction using an UP (dG) primer (10 homopolymeric G) and the inner-gene-specific primer 5GSP1 (Table 1). An extra round of PCR was conducted with a 1000-fold dilution of the original PCR product as the template using the UP primer and a nested 5GSP2 primer (Table 1) to get a pure and specific DNA product. The final product was subjected to nucleotide sequencing (BGI) using the primer 5GSP2 to identify the *ChHp1bp3* 5′ cDNA sequence.

To determine the 3′ end sequence, the previously obtained first strand cDNA pool that was reverse transcribed with the UP (dT) primer was applied as a template for PCR amplification using a UP primer and the gene-specific primer 3GSP1. The PCR product was diluted 1000-fold and used for an additional round of PCR using a poly(dA) primer and a nested 3GSP2 primer. The obtained specific DNA product was analyzed with nucleotide sequencing using the primer 3GSP2.

### Purification of Histidine-tagged *Ch*HP1BP3 in *E. coli*

The *Ch*HP1BP3 coding sequence was amplified from the *C*. *hongkongensis* cDNA pool via PCR with HP1BP3-F-NdeI and HP1BP3-R-XhoI primers. The PCR product was cloned into the pET22b(+) vector that had been digested by *Nde* I and *Xho* I to yield pET22b(+)-*ChHp1bp3*. The latter was transformed into *E*. *coli* BL21, which is a suitable host for protein expression. The resulting transformants were cultivated at 37 °C in LB medium supplemented with 100 μg/mL ampicillin until the OD_600_ reached 0.8. IPTG (isopropyl-β-D-thiogalactopyranoside) was introduced at a final concentration of 0.2 mM for induction, and the cells were incubated for an additional 6 h at 28 °C. The cells were collected by centrifugation and the cell pellet was resuspended in lysis buffer (50 mM Na_2_HPO_4_, pH 8.0, and 500 mM NaCl) and sonicated on ice to obtain a homologous solution. The cell debris was removed by centrifugation at 14,000 g at 4 °C for 20 min. The resulting cell extract was passed through a Ni-NTA Agarose column (Cat. No. 30210; Qiagen) column on a Biologic LP apparatus (Bio-Rad). The 6-histidine-tagged *Ch*HP1BP3 (His_6_-*Ch*HP1BP3) was purified to near homogeneity according to the manufacturer’s instructions.

### Electrophoretic mobility shift assay (EMSA)

The 5′ FITC-labeled *ChHsp70* promoter region (−304 to +62 relative to the predicted transcriptional start as +1) was amplified by PCR as described previously^26^. The labeled fragment was incubated with purified His_6_-*Ch*HP1BP3 at various concentrations for 25 min at room temperature in binding buffer (10 mM Tris-HCl, 50 mM KCl, and 1 mM DTT, pH 7.5). Extra reactions were completed using excess amounts of specific competitors (unlabeled *ChHsp70* promoter) as well as non-specific competitors (unlabeled irrelevant DNA probes). After incubation, the complexes were resolved on a 5% native polyacrylamide gel that was pre-run at 100 V for 30 min with running buffer (45 mM Tris-HCl, pH 8.3, 45 mM boric acid, and 10 mM EDTA) and then subsequently run for 90 min at 100 V. The FITC-labeled DNA bands were visualized by fluorescence imaging using an UVI Alliance 4.7 imager (UK).

### *Ch*HP1BP3 knockdown

Approximately 100 ml of hemolymph were aseptically withdrawn from the posterior adductor muscle of approximately one hundred oysters using 1 ml syringes equipped with 23-gauge needles. The hemocytes were collected by centrifugation at 3000 rpm for 2 min. The collected hemocytes were washed with ice-cold PBS three times and subsequently resuspended in 50 ml complete DMEM (Dulbecco’s Modified Eagle’s Medium containing 10% heat inactivated fetal bovine serum [FBS] and supplemented with 100 units/ml penicillin, 100 μg/ml streptomycin, and 0.4% NaCl). The resulting solution was plated on 24-well plates at a hemocyte density of 1 × 10^5^ cells/well. Then, the plates were incubated at 28 °C for 24 h in a humidified incubator supplied with 5% CO_2_, so as to reach 30–50% cell confluence. Then, siRNA and control RNA prepared by Sangon were individually transfected into the hemocytes using Lipofectamine^TM^ 2000 (Invitrogen) according to the manufacturer’s instructions in DMEM complete medium supplemented with 0.4% NaCl and lacking antibiotics. The cells were incubated at 28 °C for 4 h, followed by washing with PBS. The medium was shifted back to original completed DMEM supplemented with 0.4% NaCl, followed by incubation of 20 h. For heat shock treatment, the samples were incubated at 37 °C for 20 min, followed by a recovery period at 28 °C for 1 h before collecting the first sample. The collected hemocytes were then subjected to RT-PCR analysis.

### Plasmid construction

The *ChHp1bp3* coding sequence was amplified from the *C*. *hongkongensis* cDNA pool by PCR using the HP1BP3-E-KpnI and HP1BP3-E-XhoI primers (Table 1). The resulting PCR products were digested with the corresponding restriction enzymes and ligated into the pcDNA3.1 eukaryotic expression vector that had been cut with *Kpn* I and *Xho* I to yield pcDNA3.1-*ChHp1bp3*. The accuracy of fragment insertion was confirmed by sequencing (Liuhe BGI). The promoter-probe vector pGL3-*ChHsp70p* was constructed in our previous study^26^. The plasmids were kept at −20 °C until further analysis.

### Dual-luciferase reporter assay

HEK293T cells were cultivated following the procedures described above for hemocytes cultivation. A confluence percentage of 90–95% was reached after plating onto 24-well plates. The dual-luciferase reporter assay was performed as described previously^26^. Briefly, the pGL3*-ChHsp70* (200 ng/well), pRL-TK (20 ng/well, Promega), pcDNA3.1-*ChHp1bp3* (200 ng/well) or empty pcDNA3.1 (200 ng/well) plasmids were co-transfected into HEK293T cells using Lipofectamine^TM^ 2000 (Invitrogen) according to the manufacturer’s instructions in the DMEM medium lacking FBS and antibiotics. After incubation at 37 °C for 4.5 h, the medium was shifted back to complete DMEM, followed by an additional incubation for 24 h prior to collecting the first sample. A Dual-luciferase Reporter Assay System (Promega) was applied to determine the luciferase activities according to the manufacturer’s instructions. Renilla luciferase activities were used to correct for the transfection efficiency.

### Quantitative real-time PCR

Total RNA was extracted from hemocytes transfected with either siRNA or control RNA as well as from the gills of oysters that were either non-stressed or stressed by heat, CdCl_2_, or NP at various time points using the Trizol reagent (Cat. No. 15596-026; Invitrogen). Two micrograms of total RNA was used as template to synthesize the first-strand cDNA with MLV Reverse transcriptase (Promega) and oligo (dT) primer. The resulting cDNA was subsequently purified with a QIA quick column (Qiagen). Quantitative real-time PCR was performed in a 20 μL reaction mixture comprising SYBR Green Real-time PCR Master Mix (Toyobo, Japan), cDNA and one of the following gene-specific primer pairs: (1) RT-HP1BP3-Up/RT-HP1BP3-Down, (2) RT-HSP70-Up/RT-HSP70-Down or (3) Actin-Up/Actin-Down (internal control). The reaction mixtures were then subjected to the following thermal cycling conditions: (1) 95 °C for 4 min; (2) 40 cycles at 94 °C for 20 s, 62 °C for 20 s, 72 °C for 30 s; and (3) a gradient from 65 °C to 95 °C over 10 min with continuous detection. The expression of the *ChHp1bp3* and *ChHsp70* mRNAs was calculated relative to the amount of β-actin present. Negative controls were carried out without the addition of reverse transcriptase to confirm that the amplified products were derived from the mRNA transcripts instead of the genomic DNA. The assays were completed in triplicate for each sample.

### Statistical analysis

The data were analyzed using one-way ANOVA followed by the Tukey HSD test. Differences were regarded as statistically significance at *P* < 0.05. IBM SPSS Statistics 19.0 was applied for the statistical analysis.

### Nucleotide sequence accession number

The *ChHp1bp3* cDNA sequence reported in the present study was deposited in the GenBank with accession number KX981984.

## Electronic supplementary material

Supplementary data
