# Supplementary material for: The novel transcriptional factor HP1BP3 negatively regulates Hsp70 transcription in Crassostrea hongkongensis
Source: Sci Rep. 2017 May 3;7:1401. doi: 10.1038/s41598-017-01573-y (PMC5431216; doi:10.1038/s41598-017-01573-y)
Supplement: Supplementary file 1 — Supplementary data [file 41598_2017_1573_MOESM1_ESM.doc]

**The novel transcriptional factor HP1BP3 negatively regulates *Hsp70* transcription in *Crassostrea hongkongensis***

Delin Xu**^†^**, Qin Yang**^†^**, Miao Cui*, Qizhong Zhang*

* Corresponding authors

**^†^** These authors contributed equally to this work.

Department of Ecology, Institute of Hydrobiology, School of Life Science and Technology, Key Laboratory of Eutrophication and Red Tide Prevention of Guangdong Higher Education Institutes, Engineering Research Center of Tropical and Subtropical Aquatic Ecological Engineering, Ministry of Education, Jinan University, Guangzhou, 510632, PR China

**Email addresses**: [zhangqzdr@126.com](mailto:zhangqzdr@126.com) (Q. Zhang)

[cuisanshui@163.com](mailto:cuisanshui@163.com) (M. Cui)

**Tel:** (0086)-20-85225808

**Materials and Methods**

***In-gel digestion and peptide sample preparation for the identification of purified proteins***

All solvents used in this procedure were HPLC grade. Protein bands were excised and washed three times with a solution of acetonitrile/deionized water (1:1 v/v) for 10 min and dehydrated with acetonitrile. The samples were finally washed with a solution of acetonitrile/100 mM ammonium bicarbonate (1:1 v/v) and dried using a Speed-Vac. Proteins in excised gels were reduced by 10 mM Tris (2-carboxyethyl) phosphine in 0.1 M ammonium bicarbonate at 56 °C for 45 min and then alkylated with 55 mM iodoacetamide in 0.1 M ammonium bicarbonate at room temperature for 30 min. After an additional washing step, the gel pieces were dried and soaked in sequencing-grade trypsin solution (500 ng) on ice for 45 min. Then, the gel pieces were immersed in 25 mM ammonium bicarbonate (pH 8.0) at 37 °C for 14-18 hours. The resulting peptides were extracted successively for 20 minutes each with 50% acetonitrile in 25 mM ammonium bicarbonate, 50% acetonitrile in 0.5% TFA with agitation. The extracts containing digested peptides were pooled together and the solvent was evaporated under vacuum.

***Micro LC-MS/MS analysis and protein database search***

In-gel digested proteins were loaded onto fused silica capillary columns (75 μm (I.D.) x 10 cm (L.) x 360 μm (O.D.)) containing aqua C_18_ reverse-phase column material in 5 μm particle size. The column was connected with an Ekspert NanoLC 400 and a splitter system was used to make a flow rate of 400 nl/min with a gradient. Buffer A (99.9 % H_2_O and 0.1% formic acid) and buffer B (99.9 % acetonitrile and 0.1% formic acid) were used to make a 35 min gradient. The gradient profile started with 5 min of 95 % buffer A, followed by a 20 min gradient from 5 % to 45% buffer B, a 5 min gradient from 45 % to 85 % buffer B, and additional 5 min of 80 % buffer B. Eluted peptides were directly electrosprayed into an mass spectrometer (AB Sciex 6600) by applying 1.8 kV of DC voltage. A data-dependent scan consisting of one full MS scan (350-1500 m/z) and 35 data-dependent MS/MS scans were used to generate MS/MS spectra of eluted peptides. MS/MS spectra were searched against the NCBI’s *Crassostrea gigas* protein sequence database (28,967 sequences) using MASCOT 2.3.01.

***Chromatin compaction measurement***

*ChHp1bp3* was knocked down in oyster hemocytes according to the protocol described previously in this study. Cellular chromatin was extracted from the hemocytes using Chromatin Extraction Kit (Cat. No. ab117152; abcam) according to the manufacturer’s instructions. One hundred microliter chromatin isolated from each sample was mixed with 0.5 unit of MNase (Cat. No. N3755; Sigma), and the digestion reaction was started by adding 5 μl of 0.1 M CaCl_2_ solution. An incubation was allowed at 28 °C water bath for 2 min. To stop the digestion reaction, 10 μl of 0.1 M EGTA was introduced, followed by a 2 min incubation on ice. Then the samples were applied to 1 % agarose gel electrophoresis for analysis.

**Supplementary Fig. 1.**

**
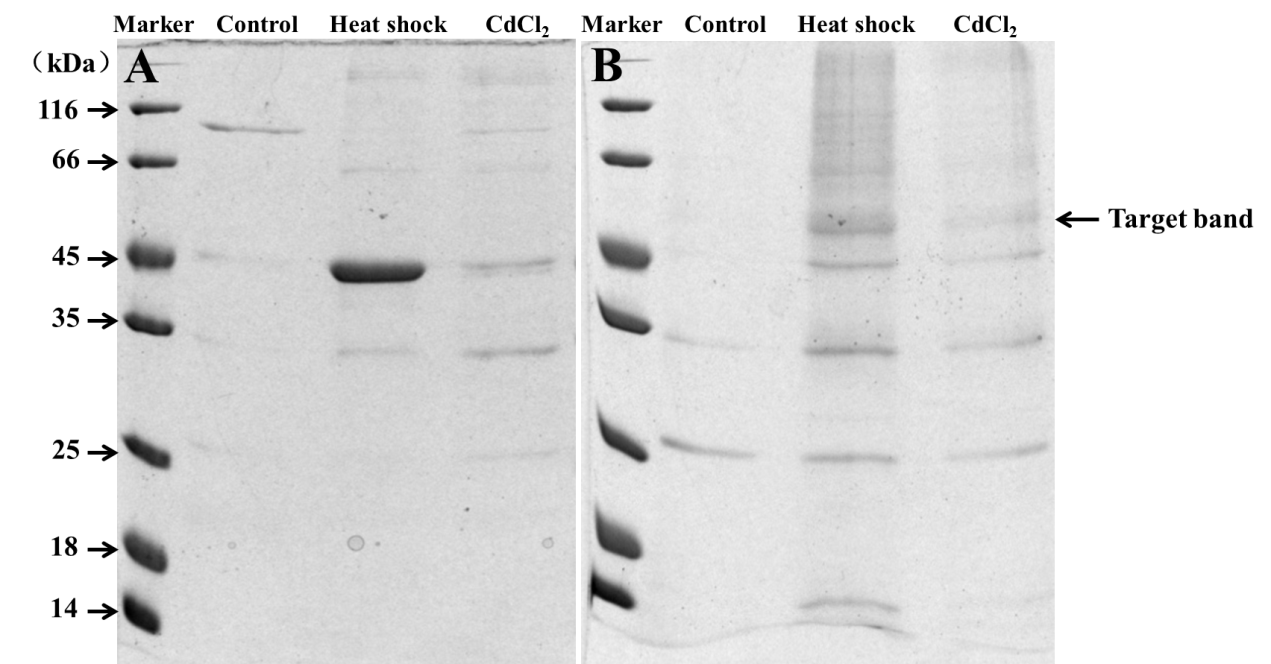
**

**Supplementary Fig. 1.** Proteins isolated by DNA affinity purification with the *ChHsp70* promoter with 15 min (A) and 30 min (B) incubations, respectively. Samples were prepared from the gills isolated from the oysters of the control, heat-shocked and CdCl_2_ treated groups. The position of the target protein band of interest was indicated by an arrow. The sizes of the molecular marker are indicated on the left-hand side in kDa.

**Supplementary Fig. 2.**


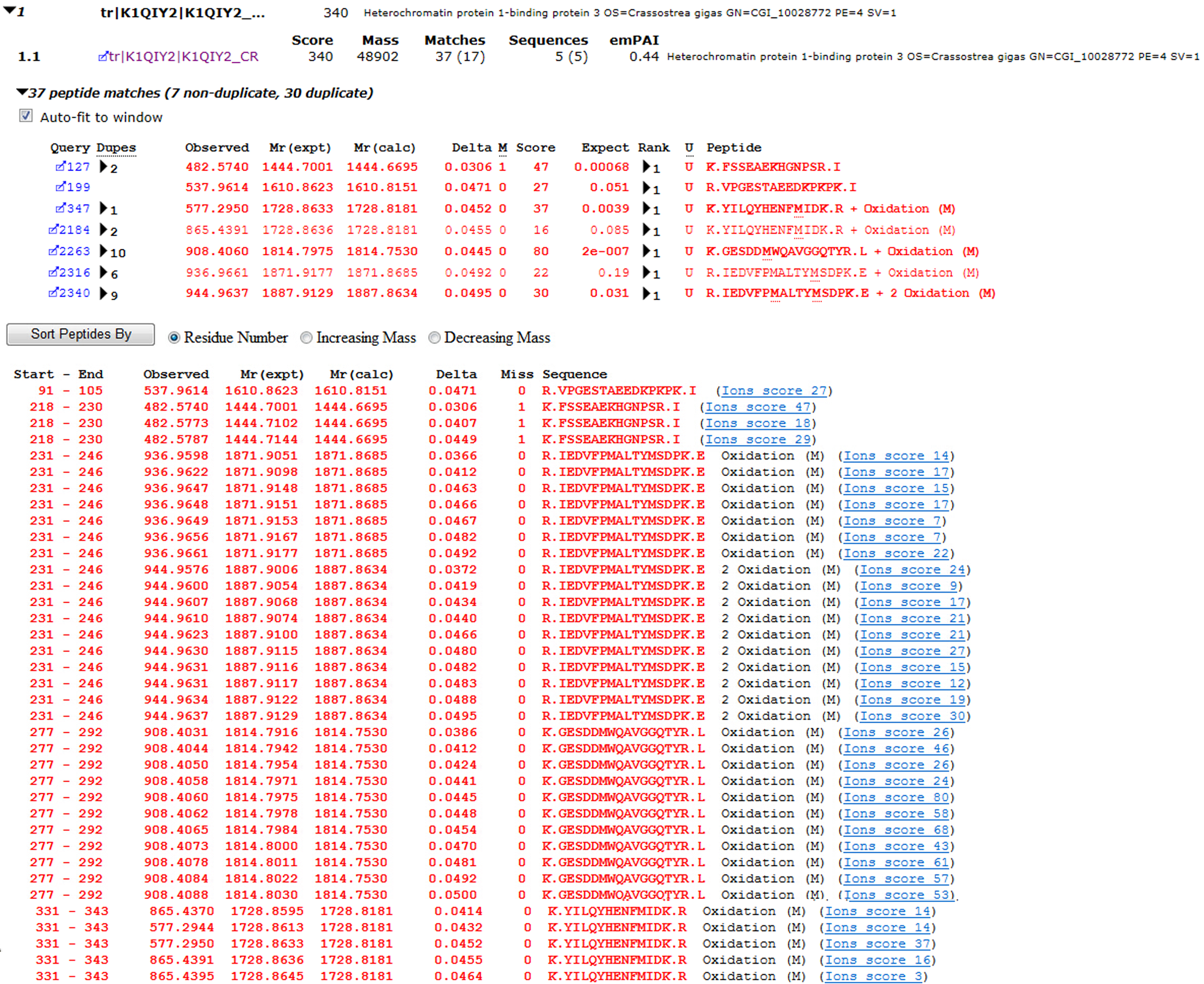


**Supplementary Fig. 2. Mass Spectrometry result of the target protein band.**

**Supplementary Fig. 3.**

**
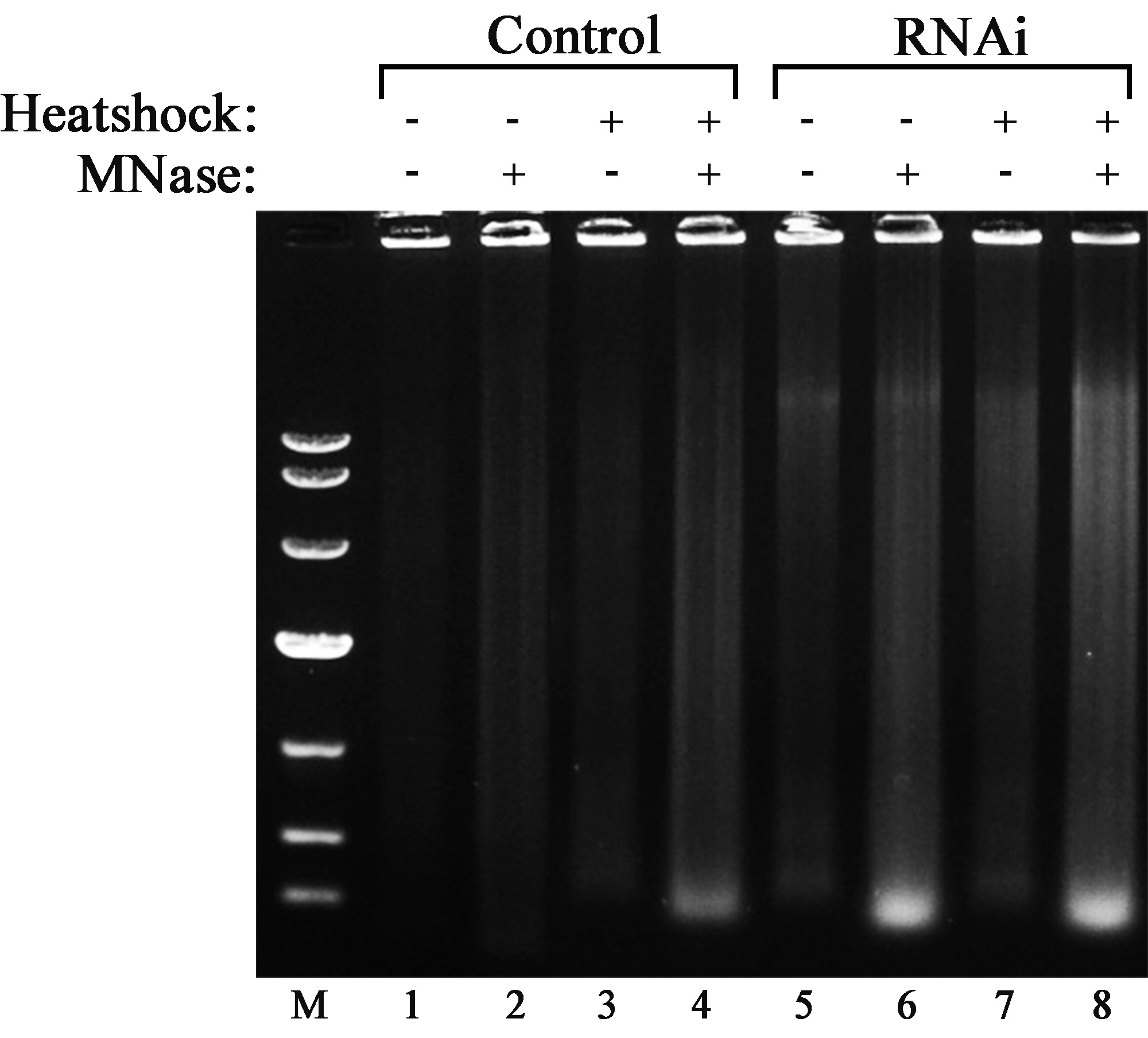
**

**Supplementary Fig. 3. *Ch*HP1BP3 depletion alters higher-order chromatin structure. Chromatin were extracted from the hemocytes samples 24 h post *ChHp1bp3* knock down.**
